# Association Between Phase Angle and AWGS 2025-Defined Sarcopenia in Community-Dwelling Older Adults

**DOI:** 10.3390/healthcare14162568

**Published:** 2026-08-17

**Authors:** Masayuki Hoshi, Yui Miyazaki, Tomoka Ogata, Koei Ishida, Yuu Okabe, Misuzu Kusano, Tatsuya Nakanowatari, Toshimi Sato, Akihiko Asao, Natsumi Kimura, Maki Ogasawara, Yuko Horikoshi, Rie Sakuraba-Hirata, Akiomi Yoshihisa, Hiroshi Hayashi, Kazuaki Iokawa, Toshimasa Sone, Yoshitaka Shiba

**Affiliations:** 1 Department of Physical Therapy, School of Health Sciences, Fukushima Medical University, Fukushima 960-8516, Japan; nakanowa@fmu.ac.jp (T.N.); satot-pt@fmu.ac.jp (T.S.); y-shiba@fmu.ac.jp (Y.S.); 2 Department of Rehabilitation, Ohtanishinouchi Hospital, Ohta General Hospital Foundation, Fukushima 963-8558, Japan; 3 Department of Rehabilitation, Ohara General Hospital, Fukushima 960-8611, Japan; 4 Department of Rehabilitation, Iwaki City Medical Center, Fukushima 973-8555, Japan; 5 Department of Rehabilitation, Jusendo Kaguyama Hospital, Yuasa Hoonkai Foundation, Fukushima 963-8833, Japan; 6 Department of Rehabilitation, Matsumura General Hospital, Iwaki Saiseikai Foundation, Fukushima 970-8516, Japan; 7 Department of Occupational Therapy, School of Health Sciences, Fukushima Medical University, Fukushima 960-8516, Japan; a-asao@fmu.ac.jp (A.A.); k-natsu@fmu.ac.jp (N.K.); oga-maki@fmu.ac.jp (M.O.); hhayashi@fmu.ac.jp (H.H.); iokawa@fmu.ac.jp (K.I.); tsone@fmu.ac.jp (T.S.); 8 Department of Clinical Laboratory Sciences, School of Health Sciences, Fukushima Medical University, Fukushima 960-8516, Japan; ykokubun@fmu.ac.jp (Y.H.); hrt@fmu.ac.jp (R.S.-H.); yoshihis@fmu.ac.jp (A.Y.)

**Keywords:** phase angle, sarcopenia, bioelectrical impedance analysis, community-dwelling older adults, physical function, AWGS 2025

## Abstract

**Highlights:**

**What are the main findings?**

- Phase angle (PhA) was significantly associated with physical function measures, including grip strength, knee extension strength, walking speed, and Timed Up and Go performance in community-dwelling older adults.
- Lower PhA values were independently associated with prevalent sarcopenia in older women, and an exploratory PhA cutoff of 4.48° was identified.

**What are the implications of the main findings?**

- PhA may be associated with physical function and prevalent sarcopenia among community-dwelling older women.
- The exploratory PhA cutoff requires validation in larger, independent cohorts and longitudinal studies before clinical application.

**Abstract:**

**Background/Objectives:** Phase angle (PhA), derived from bioelectrical impedance analysis (BIA), has been proposed as an indicator of cellular health, nutritional status, and physical function. This study aimed to investigate the association between PhA, physical function, and sarcopenia defined according to the Asian Working Group for Sarcopenia (AWGS) 2025 criteria in community-dwelling older adults. In addition, we explored a preliminary PhA cutoff for discriminating women with prevalent sarcopenia. **Methods:** This cross-sectional study included 317 participants (81 men, 236 women) aged ≥65 years who participated in functional assessments conducted in three locations of Fukushima Prefecture, Japan, in 2024. PhA at 50 kHz was measured using BIA. Assessments included body composition, grip strength, knee extension strength, maximum walking speed, Timed Up and Go (TUG), the Montreal Cognitive Assessment, Japanese version (MoCA-J), and the Kihon Checklist (KCL). Sarcopenia was defined according to the AWGS 2025 criteria. Group differences were examined using the Mann–Whitney *U* test, and associations were assessed using Spearman’s rank correlation coefficients. Age-adjusted logistic regression analysis was performed in women to examine the association between PhA and sarcopenia. Receiver operating characteristic (ROC) analysis was performed in women to explore a preliminary PhA cutoff because the limited number of men with sarcopenia precluded a stable sex-specific analysis. **Results:** Sex-specific Spearman’s rank correlation analyses showed that lower PhA values were associated with lower muscle strength (grip strength and knee extension strength) and poorer physical function (maximum walking speed and Timed Up and Go [TUG] performance) in both men and women. Lower PhA values were independently associated with prevalent sarcopenia in women after adjustment for age (odds ratio, 0.26; 95% CI, 0.09–0.80). ROC analysis identified an exploratory PhA cutoff of 4.48° for discriminating women with prevalent sarcopenia (AUC = 0.67; sensitivity = 0.65; specificity = 0.71), indicating modest discriminative performance. **Conclusions:** Lower PhA values were cross-sectionally associated with poorer physical function and prevalent sarcopenia among community-dwelling older women. An exploratory PhA cutoff of 4.48° was identified to discriminate prevalent sarcopenia in women. However, this cutoff should be considered hypothesis-generating rather than an established clinical threshold and requires validation in larger, independent cohorts and prospective longitudinal studies before clinical application.

## 1. Introduction

With the rapid aging of the global population, sarcopenia has attracted increasing attention as a major geriatric syndrome associated with disability, reduced quality of life, and increased mortality [1,2]. It is characterized by age-related loss of skeletal muscle mass accompanied by decreased muscle strength and/or physical function [2]. Aging is considered the primary cause of sarcopenia, while physical inactivity, chronic disease, inflammation, and malnutrition are recognized as important contributing factors [2]. The progression of sarcopenia is associated with increased risks of falls, fractures, hospitalization, disability, and the need for long-term care [2,3]. The prevalence of sarcopenia among Japanese older adults aged ≥65 years has been reported to be 11.5% in men and 16.7% in women [3].

Dynapenia, defined as age-related loss of muscle strength independent of muscle mass loss, has also been recognized as an important condition in older adults [4]. Similar to sarcopenia, dynapenia is more prevalent in women and has been associated with impaired physical function, falls, hospitalization, and mortality [5]. Early identification of sarcopenia is important for preventing physical disability and maintaining healthy life expectancy in older adults. Reflecting this need, the Asian Working Group for Sarcopenia 2025 (AWGS 2025 [6]) proposed updated diagnostic criteria for sarcopenia, emphasizing the combined assessment of muscle mass and muscle strength for early detection and intervention [6]. 

Phase angle (PhA), derived from bioelectrical impedance analysis (BIA), is considered an indicator of cellular membrane integrity, intracellular and extracellular fluid balance, and nutritional status [7]. Higher PhA values indicate greater cellular integrity, and healthy adults generally exhibit values ranging 5–7°, with women tending to show lower values than men [8,9]. In recent years, PhA has attracted increasing attention as a prognostic and nutritional marker and has been applied in various clinical settings, including assessment of disease severity, postoperative complications, disability, and mortality risk [10]. Furthermore, lower PhA values have been associated with incident disability [11], falls, and fear of falling [12]. In addition, previous studies have reported that patients with sarcopenia exhibit lower PhA values than non-sarcopenic individuals and that PhA may be a more sensitive indicator than handgrip strength for detecting nutritional deterioration [13]. Furthermore, recent review and meta-analysis studies have consistently demonstrated that lower PhA values are associated with sarcopenia and may provide useful information for the assessment of sarcopenia [14,15]. However, considerable heterogeneity has been reported across studies with respect to participant characteristics, ethnicity, health status, diagnostic criteria, and BIA devices [14]. In addition, PhA measurements may vary across different BIA devices and manufacturers [16,17,18].

However, the diagnosis of sarcopenia generally requires multiple assessments, including measurements of muscle strength and muscle mass. Although PhA is obtained simultaneously during routine BIA measurements used for body composition assessment, few studies have evaluated its association with sarcopenia defined according to the newly published AWGS 2025 criteria in community-dwelling older adults. Furthermore, sex-specific evidence regarding the association between PhA, physical function, and AWGS 2025-defined sarcopenia remains limited. Because PhA reflects cellular integrity and nutritional status, it may provide complementary information beyond conventional body composition measures, particularly regarding physical function and overall physiological status, which are not directly captured by skeletal muscle mass alone. Therefore, PhA is not intended to replace conventional BIA-derived measurements, such as skeletal muscle mass index (SMI), but rather to serve as an additional marker that complements the current assessment of sarcopenia under the AWGS 2025 criteria.

Therefore, this study aimed to investigate the association between PhA, physical function, and AWGS 2025-defined sarcopenia in community-dwelling older adults. In addition, we explored a potential PhA threshold for discriminating women with prevalent sarcopenia.

## 2. Materials and Methods

### 2.1. Participants

The study participants were community-dwelling older adults residing in three municipalities of Fukushima Prefecture, Japan (Fukushima City, Minamisoma City, and Kagamiishi Town), who were aged 65 years or older and able to independently attend the assessment venue. Participants were recruited through municipal publicity materials, including local newsletters. Individuals certified as requiring long-term care under the Japanese long-term care insurance system were not eligible for participation. Participants who were unable to complete the required body composition or physical function assessments were excluded from the final analysis.

**Figure 1:**
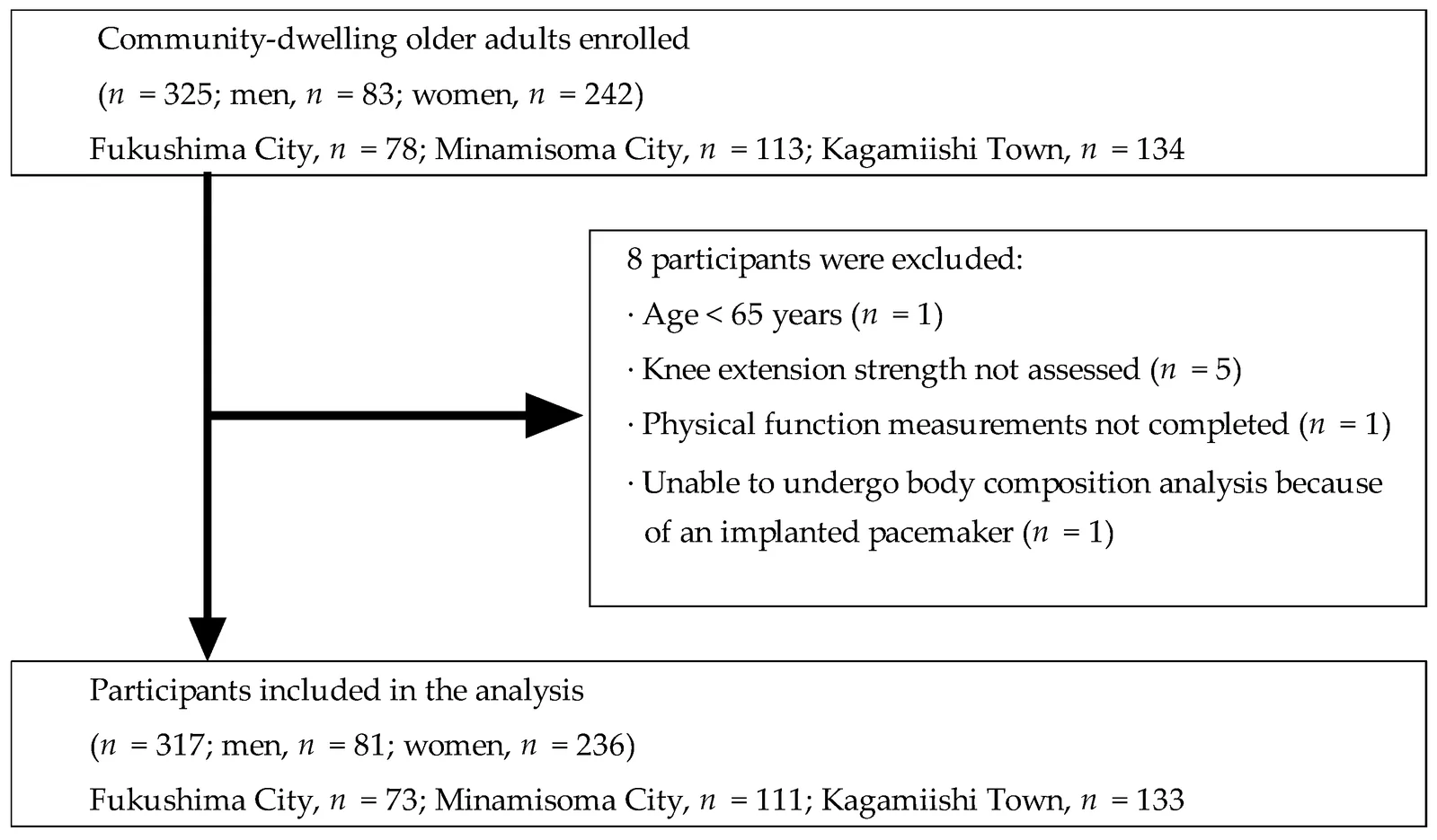
Flowchart of participant selection for the study cohort.

Of the 325 participants initially enrolled, 317 (81 men and 236 women) were included in the final analysis. Eight participants were excluded: five because knee extension strength could not be assessed, one because physical function measurements could not be completed, one because body composition analysis could not be performed owing to an implanted pacemaker, and one because the participant was younger than 65 years. Participants with incomplete data for variables required for the analyses were excluded using a complete-case approach. The participant selection process is shown in Figure 1.

### 2.2. Methods

The assessments included body composition analysis, physical function assessments (grip strength, knee extension strength, maximum walking speed, and the Timed Up and Go test [TUG]), cognitive function assessment using the Montreal Cognitive Assessment, Japanese version (MoCA-J), and questionnaires including the Kihon Checklist (KCL). Sarcopenia was assessed according to the AWGS 2025 criteria. All assessments were performed by trained examiners according to standardized procedures to ensure measurement consistency. These assessment methods have been widely used in previous studies and have demonstrated good reliability and validity in older adults.

#### 2.2.1. Body Composition Analysis

Body composition analysis was performed using an eight-point contact electrode method with a bioelectrical impedance analyzer (InBody S10; InBody Co., Ltd., Tokyo, Japan). The same InBody S10 device was used at all three assessment sites. Measurements were conducted with participants in the supine position on a bed, with electrodes attached to both thumbs, middle fingers, and ankles, according to the manufacturer’s instructions. Each measurement required approximately 100 s. The device was regularly maintained and calibrated by the manufacturer. 

The measured parameters included PhA at 50 kHz and skeletal muscle mass index (SMI). The InBody S10 estimates appendicular skeletal muscle mass using the manufacturer’s proprietary algorithm and directly provides SMI values. The SMI values displayed by the device were used in the present study in accordance with the AWGS 2025 criteria.

The InBody S10 determines impedance by measuring resistance and reactance using multifrequency currents and has been widely used for body composition assessment in clinical and research settings [19].

#### 2.2.2. Grip Strength

Participants were asked to stand with their feet slightly apart and hold a grip dynamometer (Digital Grip Dynamometer T.K.K.5401, SANKA Co., Ltd., Niigata, Japan) without allowing it to touch the body. Grip width was adjusted so that the proximal interphalangeal joints were aligned vertically when gripping. Grip strength was measured twice in the dominant hand whenever possible, and the higher value was used for analysis. When measurement of the dominant hand was not possible because of pain or upper-limb impairment, the non-dominant hand was assessed instead. All participants were able to complete grip strength testing on at least one side. Low muscle strength was defined according to the AWGS 2025 criteria.

#### 2.2.3. Knee Extension Strength

Participants sat on a chair with the knee joint flexed to 90° and their arms crossed in front of their chest, and a handheld dynamometer (μTasF-1, Anima Co., Ltd., Tokyo, Japan) sensor was placed directly above the ankle joint of the dominant leg. Maximum knee extension strength was assessed using an isometric knee extension test with a 5 s maximal contraction of the quadriceps muscle. Maximum muscle force values (N) were recorded and multiplied by the distance from the knee joint space to the sensor center to measure torque (Nm). Subsequently, muscle strength (Nm/kg) was calculated by dividing the aforementioned calculated values by the participant’s body weight. Measurements were performed twice on the dominant leg whenever possible, and the higher value was used for analysis. When participants were unable to perform a maximal contraction with the dominant leg because of pain or fatigue, the non-dominant leg was assessed instead (*n* = 2). All participants were able to complete the assessment on at least one side.

#### 2.2.4. Maximum Walking Speed

Two tape markers were placed 10 m apart on a flat floor. Additional tape markers were positioned 2 m before the starting line and 2 m beyond the finish line, resulting in a total walking distance of 14 m. Participants stood at the starting marker and were instructed to walk as fast as possible without running. Participants wore their usual closed-heel shoes whenever possible; when appropriate footwear was unavailable, suitable shoes were provided by the investigators. Participants who routinely used a walking aid were permitted to use it during the assessment, and the use of an assistive device was recorded. Two trials were performed, and the faster walking speed was used for analysis. Timing began when the leading foot crossed the 10 m start line and ended when it crossed the 10 m finish line. The time required to walk the central 10 m was recorded using a stopwatch. Maximum walking speed (m/s) was calculated by dividing the walking distance (10 m) by the recorded walking time.

#### 2.2.5. TUG

TUG time was assessed using the Hacaro Instrumented Timed Up and Go (iTUG) app (Digital Standard Co., Ltd., Osaka, Japan), which measured the time required to stand up from a seated position, walk 3 m to a marker, turn around, return to the chair, and sit down. Participants performed the test using a 40 cm-high chair. The instruction given was: “Walk as fast as possible without running.” The direction of turning was at the participant’s discretion. Participants wore their usual closed-heel shoes whenever possible; when appropriate footwear was unavailable, suitable shoes were provided by the investigators. Participants who routinely used a walking aid were permitted to use it during the assessment, and the use of an assistive device was recorded. Two trials were performed, and the shorter time was used for analysis. The Hacaro iTUG app has previously demonstrated good validity and test–retest reliability for quantitative gait assessment [20].

#### 2.2.6. Cognitive Function

The MoCA-J was used to assess cognitive function. The MoCA-J is a 30-point scale that evaluates multiple cognitive domains (visuospatial ability, executive function, attention, language, and orientation) in approximately 10 min. A score of ≤25 was defined as mild cognitive impairment (MCI) [21].

#### 2.2.7. KCL

The KCL is a self-administered questionnaire consisting of 25 yes/no items that assess functional decline in older adults. The questionnaire includes seven domains: instrumental activities of daily living (five items), physical function (five items), nutritional status (two items), oral function (three items), socialization/housebound status (two items), cognitive function (three items), and depressive mood (five items). Higher total scores indicate poorer functional status and greater frailty risk.

#### 2.2.8. Sarcopenia Assessment

Sarcopenia was diagnosed according to the AWGS 2025 criteria [6]. Participants with both low muscle strength (handgrip strength: <28 kg for men and <18 kg for women) and low skeletal muscle mass (SMI: <7.0 kg/m^2^ for men and <5.7 kg/m^2^ for women) were classified as having sarcopenia.

### 2.3. Statistical Analysis

Participants were classified into the sarcopenia and non-sarcopenia groups according to the AWGS 2025 criteria. The normality of data distribution was assessed using the Shapiro–Wilk test. Because several variables were not normally distributed, all continuous variables are presented as medians with interquartile ranges (IQRs), and comparisons between groups were performed using the Mann–Whitney *U* test. Spearman’s rank correlation coefficients were calculated to examine the associations between PhA and body composition, physical function, cognitive function, and questionnaire measures. To improve the interpretation of the non-parametric analyses, Hodges–Lehmann estimates, corresponding 95% confidence intervals, and effect sizes (*r*) for all Mann–Whitney *U* tests are provided in Supplementary Tables S1–S3.

Multivariable logistic regression analysis was performed only in women as an exploratory analysis to examine the association between PhA and sarcopenia after adjustment for age, because only seven men had sarcopenia. Odds ratios (ORs) with 95% confidence intervals (CIs) were calculated. Age was included as an adjustment variable because of its established association with sarcopenia, whereas PhA was the primary variable of interest. To reduce the risk of model overfitting given the limited number of participants with sarcopenia, the multivariable model included only age and PhA. Multicollinearity was assessed using tolerance values and variance inflation factors (VIFs). Additional variables, including study site, body size, nutritional status, physical activity, and comorbidities, were not included because the number of sarcopenia cases did not support a more complex model.

Receiver operating characteristic (ROC) curve analysis was performed to evaluate the ability of PhA to discriminate sarcopenia, and the area under the curve (AUC) with 95% confidence intervals (CIs) was calculated. The primary outcome was the association between PhA and sarcopenia in women assessed using age-adjusted logistic regression and ROC analysis. Secondary outcomes included exploratory sex-specific group comparisons and correlations between PhA and body composition, physical function, cognitive function, and questionnaire measures.

No formal a priori sample-size calculation was performed because this study was a secondary analysis of data obtained from community-based health assessment events. All eligible participants with complete data were included in the analysis. Because of the limited number of participants with sarcopenia, particularly among men, multivariable logistic regression and ROC analyses were restricted to women. The regression and ROC-derived cutoff analyses were therefore considered exploratory. Internal validation of the ROC-derived cutoff was not performed because of the limited number of participants with sarcopenia. MoCA-J and KCL scores were summarized descriptively as baseline characteristics to comprehensively characterize the study population. They were not included in the primary analyses because the primary objective of this study was to evaluate the association between PhA and AWGS 2025-defined sarcopenia rather than cognitive function or frailty status.

Statistical significance was set at *p* < 0.05. All statistical analyses were performed using SPSS (version 30; IBM Corp., Armonk, NY, USA).

### 2.4. Ethics Approval and Consent to Participate

The study protocol was reviewed and approved by the Research Ethics Committee of Fukushima Medical University (Approval No. General 2022-123). Participation in the community-based health assessment events was voluntary. Before the assessments, participants were provided with a research information sheet and received an oral explanation regarding the study, including the collection and research use of the assessment data. The study was conducted under an ethics committee-approved opt-out procedure, whereby participants were informed of the study through research information sheets and public notices issued by the organizing municipalities and were given the opportunity to decline participation or the use of their data at any time. Accordingly, the requirement for written informed consent was waived by the Research Ethics Committee.

The cognitive assessment booth, where the MoCA-J was administered, was supervised by a psychiatrist at all assessment events. If any concerns regarding a participant’s cognitive or mental status were identified during the assessment process, the psychiatrist provided an appropriate clinical evaluation and follow-up as needed. This study was conducted in accordance with the principles of the Declaration of Helsinki.

## 3. Results

### 3.1. Baseline Characteristics of the Study Participants by Sex

Table 1 presents the baseline characteristics of the study participants by sex. Men had significantly greater height, weight, grip strength, knee extension strength, PhA, and SMI than women (all *p* < 0.001), whereas no significant sex differences were observed in age, BMI, maximum walking speed, TUG performance, or KCL score. Women had significantly higher MoCA-J scores than men (*p* = 0.015).

### 3.2. Sex-Specific Comparisons Between the Sarcopenia and Non-Sarcopenia Groups

Table 2 presents sex-specific comparisons between the sarcopenia and non-sarcopenia groups according to the AWGS 2025 criteria. Among men, 7 of 81 participants (8.6%) were classified as having sarcopenia, whereas among women, 17 of 236 participants (7.2%) were classified as having sarcopenia. Among men, differences between the sarcopenia and non-sarcopenia groups were observed for most physical function and body composition measures, whereas little evidence of group differences was observed for the total KCL or MoCA-J scores. However, because only seven men met the AWGS 2025 criteria for sarcopenia, these findings should be interpreted cautiously. Estimates of the group differences, corresponding Hodges–Lehmann estimates, 95% confidence intervals, and effect sizes (*r*), are provided in Supplementary Table S2. PhA values were lower in the sarcopenia group than in the non-sarcopenia group. Among women, differences between the sarcopenia and non-sarcopenia groups were observed for height, weight, BMI, grip strength, PhA, and SMI, whereas little evidence of group differences was observed for knee extension strength, maximum walking speed, TUG, KCL, or MoCA-J scores. Estimates of the group differences, corresponding Hodges–Lehmann estimates, 95% confidence intervals, and effect sizes (*r*) are provided in Supplementary Table S3. In both men and women, PhA values were lower in the sarcopenia group than in the non-sarcopenia group.

### 3.3. Correlation Between Body Composition-Derived PhA and Physical Function

**Figure 2:**
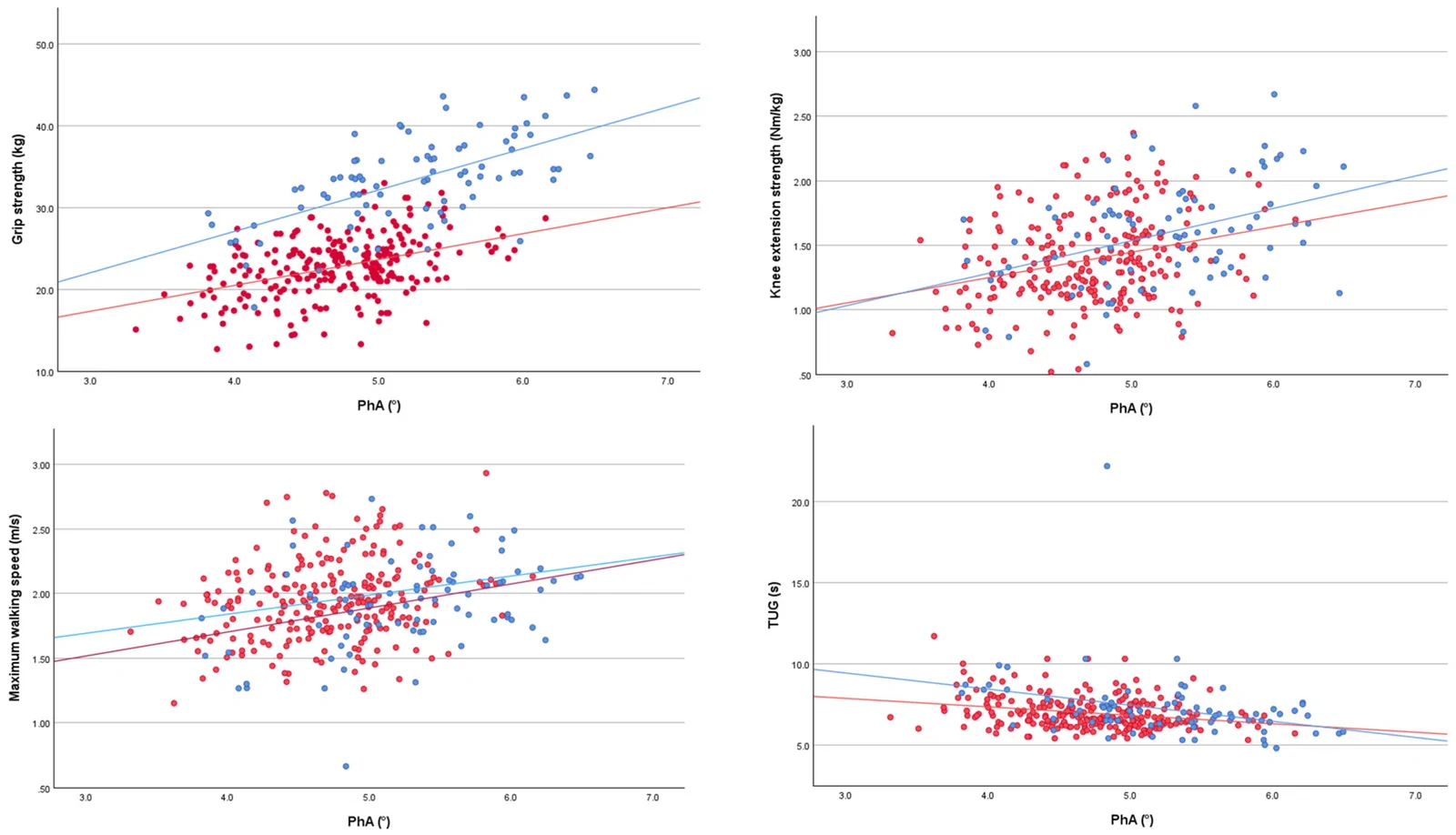
Scatter plots illustrating the associations between phase angle (PhA) and physical function measures. Male participants are shown as blue circles with blue regression lines, whereas female participants are shown as red triangles with red regression lines. Sex-specific Spearman’s rank correlation coefficients are presented in Table 3. Notes: Values are presented as Spearman’s rank correlation coefficients (*r*_s_). PhA, phase angle; TUG, Timed Up and Go.

Table 3 and Figure 2 present the associations between PhA and physical function measures. Sex-specific Spearman’s rank correlation analyses showed that PhA was positively correlated with grip strength, knee extension strength, and maximum walking speed and negatively correlated with TUG in both men and women. Detailed sex-specific correlation coefficients are presented in Table 3.

### 3.4. Multivariable Logistic Regression Analysis of Factors Associated with Sarcopenia

Table 4 presents the results of the multivariable logistic regression analysis in older women. In the model that included PhA and age, PhA was significantly associated with sarcopenia (OR = 0.26, 95% CI: 0.09–0.80, *p* < 0.05), whereas age was not (OR = 0.99, 95% CI: 0.89–1.10, *p* = 0.80). 

### 3.5. Exploratory Use of PhA for Sarcopenia Screening in Older Women

**Figure 3:**
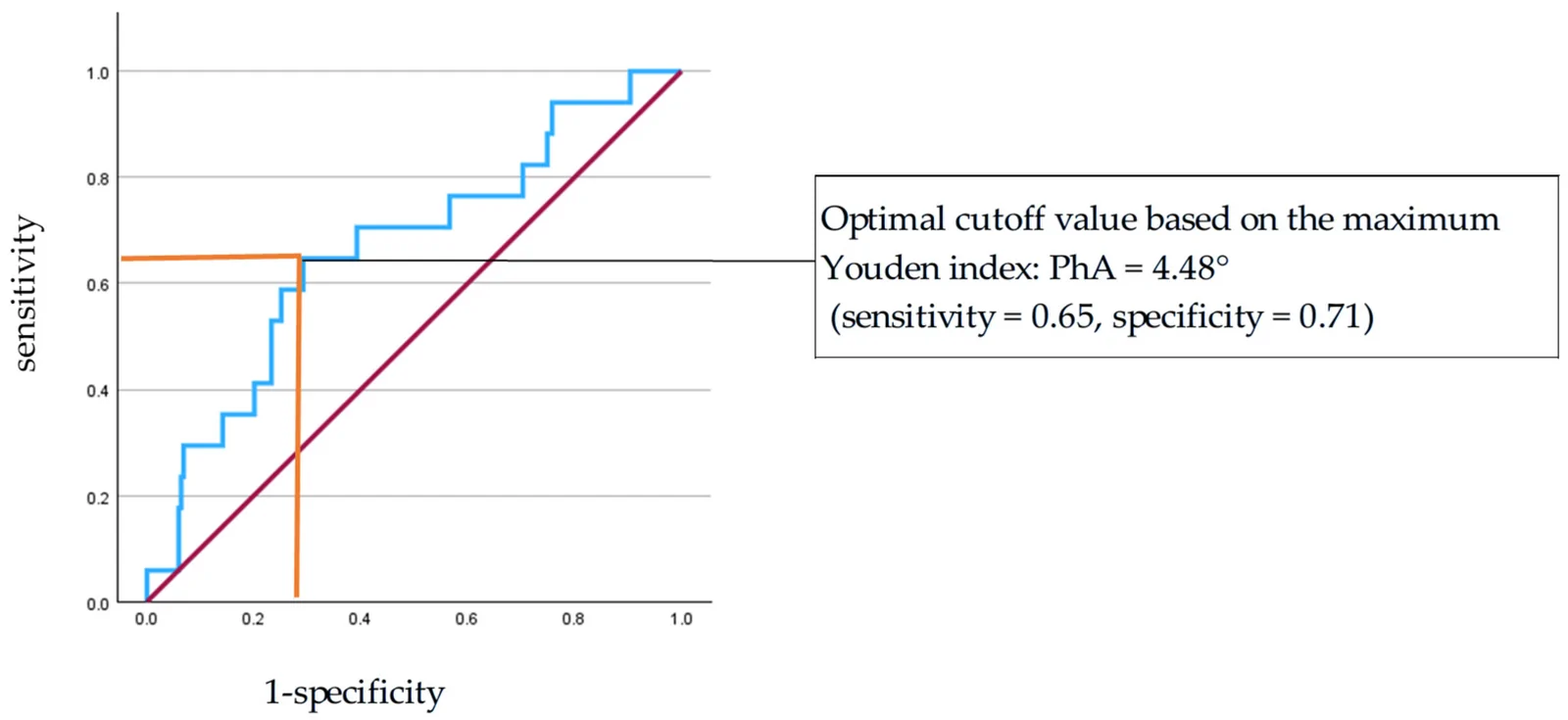
ROC curve of PhA for discriminating sarcopenia in older women. This ROC analysis was exploratory and based on 17 women with sarcopenia. The proposed cutoff should be considered hypothesis-generating and requires validation in independent cohorts. Notes: The blue line represents the ROC curve, the red line represents the reference line (AUC = 0.5). The intersection of the orange lines indicates the optimal cutoff value based on the maximum Youden index.

Figure 3 shows the ROC curve for PhA (50 kHz) for discriminating sarcopenia among older women. ROC analysis yielded an AUC of 0.67 (95% CI: 0.53–0.81, *p* < 0.05), indicating modest discriminative ability. In this exploratory analysis, the maximum Youden index identified a PhA value of 4.48° as the optimal cutoff, with a sensitivity of 0.65 and specificity of 0.71. Because this cutoff was derived from a relatively small number of women with sarcopenia (n = 17) without internal validation, it should be regarded as a hypothesis-generating estimate and interpreted with caution.

## 4. Discussion

The present study investigated the association between PhA, physical function, and AWGS 2025-defined sarcopenia in 317 community-dwelling older adults aged ≥65 years. PhA showed moderate positive correlations with handgrip strength and knee extension strength, weak positive correlations with maximum walking speed, and a weak negative correlation with TUG performance. These findings support an association between PhA and physical function in community-dwelling older adults and suggest that PhA may provide complementary information regarding sarcopenia and age-related functional decline.

The observed associations between PhA and measures of muscle strength and mobility in the present study are consistent with previous findings. PhA has been shown to decline with age and correlate with physical function [22], physical activity levels [23], and nutritional status [24]. Furthermore, lower PhA values have been linked to impaired cellular integrity and disease-related malnutrition [25,26]. 

Recent reviews and meta-analyses have consistently reported an association between lower PhA values and sarcopenia and highlighted the potential utility of PhA as a supportive parameter for sarcopenia assessment [14,15]. Zhang et al. reported that PhA demonstrated moderate diagnostic performance for identifying individuals at risk of sarcopenia, with pooled sensitivity and specificity of approximately 80% and 70%, respectively. However, substantial heterogeneity was observed among studies, and factors such as ethnicity, body mass index, health status, diagnostic criteria, and BIA devices were identified as important sources of variability. Therefore, further investigations in different populations using updated diagnostic criteria remain warranted. 

The diagnostic performance observed in the present study (AUC = 0.67) was modest and lower than that reported by Zhang et al. in their recent meta-analysis. Several factors may explain this difference. First, our participants were community-dwelling older adults who voluntarily attended community health assessment events and generally exhibited relatively good physical function, resulting in a narrower functional range and a lower prevalence of sarcopenia than in studies including hospitalized or clinical populations. Second, sarcopenia was diagnosed according to the recently updated AWGS 2025 criteria, which may not be directly comparable with earlier studies included in the meta-analysis. Third, PhA values are influenced by the type of BIA device and measurement protocol, which may contribute to between-study variability. Finally, the relatively small number of women with sarcopenia (n = 17) limited the precision of the ROC analysis and may have contributed to the modest discriminative performance observed in this study.

In the present study, a PhA value of 4.48°was identified as a hypothesis-generating estimate for discriminating sarcopenia in older women. In contrast, a previous study reported a cutoff value of 3.55° for identifying sarcopenia and dynapenia in women with a mean age in their 80s [27]. The higher value observed in the present study may be attributable to differences in participant characteristics, age distribution, and the use of the AWGS 2025 criteria for sarcopenia diagnosis. These findings are consistent with previous reports indicating that PhA thresholds vary according to study population characteristics and assessment methods. Therefore, the cutoff identified in this study should not be regarded as an established clinical threshold but rather as a preliminary, hypothesis-generating estimate that requires validation in larger, independent populations before clinical application. In our previous study [28], which investigated pre-frailty screening in community-dwelling older women, the PhA cutoff ranged from 4.19° to 4.74°. The cutoff identified in the present study (4.48°) fell within this previously reported range. Although this overlap suggests that similar PhA cutoff values may be identified across studies of pre-frailty and sarcopenia, the present cross-sectional data do not allow conclusions regarding a physiological continuum or progression from pre-frailty to sarcopenia. Therefore, this observation should be regarded as a hypothesis-generating finding that warrants further investigation in longitudinal studies. Both studies were exploratory and involved relatively small numbers of outcome events. Therefore, these findings should be interpreted with caution, and validation in larger, independent cohorts is required.

Although lower PhA values were observed in participants with sarcopenia in both women and men, the findings in men should be interpreted with caution because only seven male participants were classified as having sarcopenia. Sex-related differences in body composition may partially contribute to differences in PhA values between women and men. In addition, the relatively small number of men with sarcopenia may have reduced model stability and resulted in unstable estimates. In addition, the relatively small number of men with sarcopenia may have reduced model stability and resulted in unstable estimates.

PhA can be measured rapidly, noninvasively, and conveniently using BIA devices, making it suitable for use not only in clinical settings but also in community health assessment programs. In addition to its association with physical function and nutritional status, recent studies have suggested that PhA may also be associated with cognitive function and cognitive decline [13]. Among community-dwelling older women, the present findings suggest that lower PhA values are associated with sarcopenia and age-related functional decline. However, longitudinal and intervention studies are needed to determine whether repeated PhA assessment has predictive value or can support decisions regarding further evaluation or intervention. The clinical utility of PhA for identifying sarcopenia in older men also remains uncertain and should be investigated in larger studies.

There are several limitations to the present study. First, participants were recruited from only three municipalities in Fukushima Prefecture through voluntary participation and were able to attend the assessment venue independently. Individuals with long-term care certification or implanted electronic devices were excluded. Consequently, the study population likely consisted of relatively healthy and mobile community-dwelling older adults, introducing the possibility of selection and spectrum bias. Therefore, the findings may not be generalizable to frailer older adults or clinical populations. Furthermore, because data from three municipalities were pooled for analysis, potential site-specific differences were not formally examined.

Second, because this study was conducted as part of community-based health assessment events, pre-measurement conditions, including fasting status, recent food and fluid intake, bladder emptying, recent exercise, alcohol intake, and room conditions, were not standardized. In addition, grip strength was measured primarily in the dominant hand rather than using the maximum value obtained from both hands. Furthermore, PhA values were obtained using the InBody S10, and previous studies have reported inter-device variability in PhA measurements. Therefore, this cutoff value is most directly applicable to assessments using the InBody S10, and comparability with other BIA devices should be verified in future studies.

Third, the relatively small number of participants with sarcopenia, particularly men, limited statistical power and model stability. Consequently, only age was included as an adjustment variable, and additional potential confounders could not be incorporated into the multivariable model. Furthermore, several sex-specific group comparisons and correlation analyses were conducted as secondary analyses without formal correction for multiple testing. Therefore, some statistically significant findings may represent false-positive results and should be interpreted with caution. The ROC-derived cutoff was based on only 17 women with sarcopenia and was developed and evaluated using the same dataset without internal validation. Accordingly, the proposed cutoff should be regarded as a hypothesis-generating estimate rather than an established clinical screening threshold and requires validation in larger, independent cohorts before clinical application. Multicollinearity was assessed using tolerance values and variance inflation factors (VIFs), and no evidence of multicollinearity was observed. However, because of the limited number of participants with sarcopenia, comprehensive logistic regression model diagnostics, including assessment of the linearity of the logit, calibration, and influential observations, were not performed. Therefore, the regression findings should be interpreted as exploratory.

Fourth, both PhA and skeletal muscle mass index were obtained using the same BIA device. Therefore, the observed association between PhA and sarcopenia may partially reflect shared measurement characteristics rather than a fully independent physiological relationship. Future studies should confirm these findings using complementary methods for assessing muscle mass and cellular health. Finally, because this study employed a cross-sectional design, causal or temporal relationships between PhA and sarcopenia cannot be established. Longitudinal studies are needed to determine whether changes in PhA are associated with the onset and progression of sarcopenia.

## 5. Conclusions

In this cohort of community-dwelling older adults, PhA derived from BIA was cross-sectionally associated with physical function and AWGS 2025-defined prevalent sarcopenia. Lower PhA values were associated with reduced muscle strength, impaired mobility, and the presence of sarcopenia, particularly among older women. Among older women, a PhA value of 4.48° was identified as a preliminary, hypothesis-generating estimate for discriminating sarcopenia. However, this exploratory cutoff should not be regarded as an established clinical threshold and requires validation in larger, independent cohorts before clinical application. Because PhA measurements may vary according to device characteristics and the present findings were derived from a relatively limited cohort, further longitudinal studies are required to validate these findings and determine whether changes in PhA are associated with the development and progression of sarcopenia.

## Figures and Tables

**Table 1:** Baseline characteristics of the study participants by sex.

	All		Men		Women		*p* Value
*n*	317		81		236		
Age (years)	75.0	(72.0–79.0)	76.0	(72.0–80.0)	75.0	(72.0–79.0)	0.343
Height (cm)	153.1	(148.2–159.5)	162.9	(159.5–167.4)	151.0	(146.9–154.5)	<0.001
Weight (kg)	53.8	(47.9–60.5)	62.0	(56.4–68.2)	51.6	(46.5–56.5)	<0.001
BMI (kg/m^2^)	22.9	(20.7–25.1)	23.8	(21.8–25.5)	22.7	(20.4–24.8)	0.07
Grip strength (kg)	24.3	(21.4–28.8)	33.6	(30.0–36.7)	22.9	(20.8–24.9)	<0.001
Knee extension strength (Nm/kg)	1.40	(1.17–1.69)	1.60	(1.32–1.83)	1.36	(1.15–1.60)	<0.001
Maximum walking speed (m/s)	1.94	(1.73–2.12)	1.95	(1.76–2.13)	1.93	(1.73–2.12)	0.854
TUG (s)	6.8	(6.3–7.5)	6.9	(6.4–7.6)	6.8	(6.3–7.5)	0.487
KCL score (points)	5.0	(3.0–7.0)	5.0	(3.0–6.0)	4.0	(3.0–7.0)	0.849
MoCA-J (points)	25.0	(22.0–27.0)	25.0	(21.0–26.0)	25.0	(22.0–28.0)	0.015
Body composition: PhA (°)	4.85	(4.44–5.19)	5.34	(4.83–5.70)	4.73	(4.39–5.04)	<0.001
Body composition: SMI (kg/m^2^)	6.3	(5.8–7.2)	7.7	(7.2–8.3)	6.0	(5.7–6.5)	<0.001

Continuous variables are presented as medians (interquartile ranges [IQRs]). BMI, body mass index; TUG, Timed Up and Go; KCL, Kihon Checklist; MoCA-J, Japanese version of the Montreal Cognitive Assessment; PhA, phase angle; SMI, skeletal muscle mass index.

**Table 2:** Sex-specific comparisons between the sarcopenia and non-sarcopenia groups.

	Men					Women				
	Non-Sarcopenia		Sarcopenia		*p* Value	Non-Sarcopenia		Sarcopenia		*p* Value
*n*	74		7			219		17		
Age (years)	75.0	(71.8–79.0)	82.0	(79.0–88.0)	0.001	75.0	(72.0–79.0)	75.0	(72.0–81.0)	0.644
Height (cm)	163.7	(160.1–167.9)	155.9	(151.0–160.0)	<0.001	151.1	(147.1–154.9)	146.9	(142.9–150.5)	0.005
Weight (kg)	63.4	(58.2–69.8)	51.5	(49.4–54.9)	0.001	52.4	(47.1–56.8)	44.2	(39.7–48.5)	<0.001
BMI (kg/m^2^)	23.9	(22.0–25.6)	20.3	(19.5–25.0)	0.034	23.0	(20.6–24.9)	20.0	(18.2–23.1)	0.006
Grip strength (kg)	33.8	(31.3–37.3)	25.7	(24.9–27.8)	<0.001	23.2	(21.3–25.2)	16.1	(14.8–17.1)	<0.001
Knee extension strength (Nm/kg)	1.63	(1.38–1.85)	1.23	(0.84–1.33)	0.001	1.36	(1.15–1.59)	1.40	(1.01–1.67)	0.912
Maximum walking speed (m/s)	2.00	(1.78–2.15)	1.54	(1.30–1.88)	0.004	1.93	(1.73–2.12)	1.93	(1.69–2.05)	0.667
TUG (s)	6.8	(6.4–7.5)	8.4	(7.1–8.7)	0.005	6.7	(6.2–7.5)	6.9	(6.6–7.6)	0.354
KCL score (points)	5.0	(3.0–6.3)	4.0	(2.0–6.0)	0.806	4.0	(3.0–7.0)	4.0	(3.0–7.0)	0.751
MoCA-J (points)	25.0	(21.0–26.0)	22.0	(20.0–25.0)	0.136	25.0	(22.0–28.0)	25.0	(21.0–28.0)	0.626
Body composition: PhA (°)	5.36	(4.85–5.71)	4.13	(3.99–4.49)	<0.001	4.74	(4.41–5.05)	4.40	(3.97–4.94)	0.023
Body composition: SMI (kg/m^2^)	7.7	(7.2–8.3)	6.2	(6.0–6.4)	<0.001	6.1	(5.8–6.5)	5.1	(4.8–5.5)	< 0.001

Continuous variables are presented as medians (interquartile ranges [IQRs]). BMI, body mass index; TUG, Timed Up and Go; KCL, Kihon Checklist; MoCA-J, Japanese version of the Montreal Cognitive Assessment; PhA, phase angle; SMI, skeletal muscle mass index.

**Table 3:** Sex-specific Spearman’s rank correlations between phase angle and physical function measures.

Physical Function Measure	Men (*n* = 81)		Women (*n* = 236)	
	*r*_s_	*p* Value	*r*_s_	*p* Value
Grip strength (kg)	0.59	<0.001	0.39	<0.001
Knee extension strength (Nm/kg)	0.37	0.001	0.27	<0.001
Maximum walking speed (m/s)	0.35	0.001	0.22	0.001
TUG (s)	−0.42	<0.001	−0.23	<0.001

Notes: Data are presented as sex-specific Spearman’s rank correlation coefficients (*r*_s_). TUG, Timed Up and Go.

**Table 4:** Multivariable logistic regression analysis of factors associated with sarcopenia in older women.

Variable	OR (95% CI)	*p* Value
PhA (per 1° increase)	0.26 (0.09–0.80)	0.02
Age (per 1-year increase)	0.99 (0.89–1.10)	0.80

OR, odds ratio; CI, confidence interval; PhA, phase angle. The dependent variable was sarcopenia (yes/no). The model was adjusted for phase angle and age.

## Data Availability

The datasets generated and/or analyzed during the current study are not publicly available due to ethical and privacy considerations related to participant confidentiality but are available from the corresponding author upon reasonable request.

## Supplementary Materials

The following supporting information can be downloaded at https://www.mdpi.com/article/10.3390/healthcare14162568/s1, Table S1. Additional statistics for Mann–Whitney U tests comparing men and women. Table S2. Additional statistics for Mann–Whitney U tests comparing men with and without sarcopenia. Table S3. Additional statistics for Mann–Whitney U tests comparing women with and without sarcopenia.
