# Supplementary material for: Association Between Phase Angle and AWGS 2025-Defined Sarcopenia in Community-Dwelling Older Adults
Source: Healthcare (Basel). 2026 Aug 17;14(16):2568. doi: 10.3390/healthcare14162568 (PMC13512330; doi:10.3390/healthcare14162568)
Supplement: Supplementary file 1 [file healthcare-14-02568-s001.zip › healthcare-4453295-supplementary.pdf]

Supplementary Table S1. Additional statistics for Mann–Whitney U tests comparing men and women.

Variable	*p* value	Hodges–Lehmann median difference	95% CI	Effect size (*r*)
Age (years)	0.343	1.0	− 1.0 to 2.0	0.05
Height (cm)	< 0.001	12.5	10.9 to 13.9	0.66
Weight (kg)	< 0.001	10.7	8.6 to 12.9	0.48
BMI (kg/m^2)	0.07	0.7	− 0.1 to 1.5	0.1
Grip strength (kg)	< 0.001	10.7	9.5 to 11.8	0.68
Knee extension strength (Nm/kg)	< 0.001	0.21	0.11 to 0.30	0.23
Maximum walking speed (m/s)	0.854	0.008	− 0.074 to 0.087	0.01
TUG (s)	0.487	0.1	− 0.20 to 0.30	0.04
KCL score	0.849	0.0	− 1.0 to 1.0	0.01
MoCA-J score	0.015	− 1.0	− 2.0 to 0.0	0.14
PhA (°)	< 0.001	0.54	0.387 to 0.699	0.36
SMI (kg/m^2)	< 0.001	1.5	1.3 to 1.7	0.62

Notes: Hodges–Lehmann estimates represent the median difference between men and women. Effect size (r) was calculated as the absolute value of the standardized Mann–Whitney test statistic divided by the square root of the total sample size ($r = |Z|/\sqrt{N}$; N = 317). CI, confidence interval; BMI, body mass index; KCL, Kihon Checklist; MoCA-J, Japanese version of the Montreal Cognitive Assessment; PhA, phase angle; SMI, skeletal muscle mass index; TUG, Timed Up and Go.

Supplementary Table S2. Additional statistics for Mann–Whitney U tests comparing men with and without sarcopenia.

Variable	*p* value	Hodges–Lehmann median difference	95% CI	Effect size (*r*)
Age (years)	0.001	−8.0	−12.0 to −4.0	0.36
Height (cm)	< 0.001	7.9	3.7 to 12.3	0.38
Weight (kg)	0.001	11.4	5.6 to 18.8	0.36
BMI (kg/m²)	0.034	2.45	0.30 to 4.80	0.24
Grip strength (kg)	< 0.001	8.6	5.7 to 12.3	0.43
Knee extension strength (Nm/kg)	0.001	0.479	0.250 to 0.780	0.36
Maximum walking speed (m/s)	0.004	0.407	0.157 to 0.626	0.32
TUG (s)	0.005	−1.50	−2.20 to −0.50	0.31
KCL score	0.806	0.0	−2.0 to 3.0	0.03
MoCA-J score	0.136	2.0	−1.0 to 4.0	0.17
PhA (°)	< 0.001	1.068	0.645 to 1.493	0.39
SMI (kg/m²)	< 0.001	1.6	1.10 to 2.20	0.46

Notes: Hodges–Lehmann estimates represent the median difference between the non-sarcopenia and sarcopenia groups in men. Effect size (r) was calculated as the absolute value of the standardized Mann–Whitney test statistic divided by the square root of the total sample size ($r = |Z|/\sqrt{N}$; N = 81). CI, confidence interval; BMI, body mass index; KCL, Kihon Checklist; MoCA-J, Japanese version of the Montreal Cognitive Assessment; PhA, phase angle; SMI, skeletal muscle mass index; TUG, Timed Up and Go.

Supplementary Table S3. Additional statistics for Mann–Whitney U tests comparing women with and without sarcopenia

Variable	*p* value	Hodges–Lehmann median difference	95% CI	Effect size (*r*)
Age (years)	0.644	− 1.0	− 4.0 to 2.0	0.03
Height (cm)	0.005	4.3	1.5 to 7.3	0.18
Weight (kg)	< 0.001	8.0	4.5 to 11.6	0.27
BMI (kg/m^2)	0.006	2.3	0.7 to 4.0	0.18
Grip strength (kg)	< 0.001	7.1	5.9 to 8.4	0.42
Knee extension strength (Nm/kg)	0.912	− 0.01	− 0.20 to 0.21	0.01
Maximum walking speed (m/s)	0.667	0.03	− 0.118 to 0.180	0.03
TUG (s)	0.354	− 0.20	− 0.60 to 0.20	0.06
KCL score	0.751	0.0	− 1.0 to 2.0	0.02
MoCA-J score	0.626	0.0	− 1.0 to 3.0	0.03
PhA (°)	0.023	0.314	0.042 to 0.585	0.15
SMI (kg/m^2)	< 0.001	1.1	0.8 to 1.3	0.39

Notes: Hodges–Lehmann estimates represent the median difference between the non-sarcopenia and sarcopenia groups in women. Effect size (r) was calculated as the absolute value of the standardized Mann–Whitney test statistic divided by the square root of the total sample size ($r = |Z|/\sqrt{N}$; N = 236). CI, confidence interval; BMI, body mass index; KCL, Kihon Checklist; MoCA-J, Japanese version of the Montreal Cognitive Assessment; PhA, phase angle; SMI, skeletal muscle mass index; TUG, Timed Up and Go.
